# Severe aortic valve stenosis in low-risk elderly patients, which is the role of surgery

**DOI:** 10.1093/icvts/ivac109

**Published:** 2023-01-16

**Authors:** Nikolaos G Baikoussis, Loukia Alexopoulou-Prounia, Dimitrios Limperiadis

**Affiliations:** Cardiac Surgery Department, Ippokrateio General Hospital of Athens, Athens, Greece; Cardiac Surgery Department, Ippokrateio General Hospital of Athens, Athens, Greece; Cardiac Surgery Department, Ippokrateio General Hospital of Athens, Athens, Greece

**Keywords:** Aortic valve stenosis, Aortic valve replacement, Transcatheter valve implantation, Heart valve surgery

We read with great interest the paper published by Magro and Sousa-Uva [1] on the superior technique in terms of reported composite outcomes and survival, among transcatheter aortic valve replacement (TAVR) and surgical aortic valve replacement for low-risk patients aged >70–75 years with severe aortic stenosis.

It is currently unclear if older age should be a criterion for the choice between TAVR and surgical aortic valve replacement in otherwise low-risk patients. As the authors acknowledge, ‘the only low-risk randomized control trial to date regarding an elderly population (NOTION) [2, 3] did not show a statistically significant difference between the 2 approaches regarding the composite end-point of death, stroke or myocardial infarction’, while in other studies [4] subgroup analysis of the elderly patients (>75 years) also concluded in similar results for both techniques regarding death or stroke at 2 years follow-up.

As the authors acknowledge, risk scores and other procedural risk factors associated or not with age comprise the cornerstone of approach selection. We therefore agree with the decision of the authors to review the current literature to propose the best management option for your patients and also elucidate future heart-team discussions.

While we support the conclusions, we would like to point out that the main limitation of all studies included in the review is the fact that they were not randomized, apart from one, and therefore subject to a significant source of bias. What is more, as the authors acknowledge, since PARTNER 2 trial’s 5-year results showed a significantly higher rate of mild paravalvular aortic regurgitation (33.3% vs 6.3%), frequency of hospitalizations (33.3% vs 25.2%) and aortic valve reinterventions (3.2% vs 0.8%) [1], primary composite end points and the limited follow-up period of the existing studies may not be enough to justify the age as an independent decision-making factor.

We agree with the authors that the current literature does not show any significant superiority in either technique; however, we believe that large RCT will solve the question as to which procedure is superior in this specific population, especially since the majority of the current studies, the group sample for 5-year follow-up outcomes is undersized, and the subgroup of patients older than 74 years old is even more undersized.

We also like to notice from our experience the importance of the type of valves used for TAVR as well as the interventional cardiologist’s skill and familiarization with each valve. Both the CoreValve^®^ Revalving system (Medtronic, Inc., Minneapolis, MN, USA) and the Edwards Sapien system (Edwards Lifesciences Corporation, Irvine, CA, USA) have proved their effectiveness in the recent outcomes of the CoreValve US Pivotal and Partner trials, respectively.

Additional RCTs are required not only to inform practice but also to grade the quality of the evidence and to update our guidelines.
